# Early clearance of Chikungunya virus in children is associated with a strong innate immune response

**DOI:** 10.1038/srep26097

**Published:** 2016-05-16

**Authors:** Diane Simarmata, David Chun Ern Ng, Yiu-Wing Kam, Bernett Lee, Magdline Sia Henry Sum, Zhisheng Her, Angela Chow, Yee-Sin Leo, Jane Cardosa, David Perera, Mong H. Ooi, Lisa F. P. Ng

**Affiliations:** 1 Singapore Immunology Network, Agency for Science, Technology and Research (A*STAR), Singapore 138648, Singapore; 2 Department of Paediatrics, Sarawak General Hospital, Kuching 93587, Sarawak, Malaysia; 3 Institute of Health & Community Medicine, Universiti Malaysia Sarawak (UNIMAS), Kota Samarahan 94300, Malaysia; 4 Institute of Infectious Disease and Epidemiology (IIDE), Tan Tock Seng Hospital, Singapore 308433, Singapore; 5 Department of Paediatrics, Sibu Hospital, Sibu 96000, Malaysia

## Abstract

Chikungunya fever (CHIKF) is a global infectious disease which can affect a wide range of age groups. The pathological and immunological response upon Chikungunya virus (CHIKV) infection have been reported over the last few years. However, the clinical profile and immune response upon CHIKV infection in children remain largely unknown. In this study, we analyzed the clinical and immunological response, focusing on the cytokine/chemokine profile in a CHIKV-infected pediatric cohort from Sarawak, Malaysia. Unique immune mediators triggered upon CHIKV infection were identified through meta-analysis of the immune signatures between this pediatric group and cohorts from previous outbreaks. The data generated from this study revealed that a broad spectrum of cytokines/chemokines is up-regulated in a sub-group of virus-infected children stratified according to their viremic status during hospitalization. Furthermore, different immune mediator profiles (the levels of pro-inflammatory cytokines, chemokines and growth and other factors) were observed between children and adults. This study gives an important insight to understand the immune response of CHIKV infection in children and would aid in the development of better prognostics and clinical management for children.

Chikungunya fever (CHIKF) is an acute illness caused by the alphavirus Chikungunya virus (CHIKV). Transmitted by *Aedes* mosquitoes, clinical manifestations include an abrupt high fever, rashes, myalgia and arthralgia that will appear after an incubation period of 3–7 days. However, chronic and incapacitating arthralgia can persist for months to years^1^. CHIKV was first identified in 1953 during a dengue-like epidemic in Tanzania, and outbreaks of CHIKF have been documented in Africa and South East Asia throughout 1960s–1990s^1^,^2^,^3^. Since 2005, CHIKV has caused outbreaks of an unprecedented magnitude in La Réunion with more than a third of its population infected^4^,^5^. Over 1.4 million cases have also occurred in India, South East Asia, and islands in the Pacific between 2006 and 2012^6^,^7^,^8^,^9^. CHIKV has since spread to the Caribbean islands in late 2013^10^, and as of November 2015, 8,275 laboratory-confirmed cases have been reported from these areas (http://www.cdc.gov/chikungunya/geo/united-states-2015.html). With the worldwide increased spread of the *Aedes* mosquitoes, CHIKV remains a major threat to public health. Thus, with no available licensed vaccine or specific treatments, CHIKV can present a social and economic burden to affected communities.

Studies on CHIKF pathogenesis have revealed that CHIKV infection induced a wide range of cytokines, chemokines and growth factors^11^,^12^. Higher levels of IL-1β and IL-6, and lower level of RANTES were also linked to severe CHIKF^11^. Systematic meta-analysis has also revealed the immune signatures in patients from different CHIKF cohorts during the 2007–2010 outbreaks across different geographic locations^13^. CHIKV infection affects all age groups. However, studies on CHIKV-induced immune mediators across cohorts have been limited to adult patients. Although CHIKV infections in children have been reported^14^,^15^,^16^, documentation remained limited to clinical descriptions with no information on the immune response. Symptoms such as high fever, rash, seizures, and weight loss were reported in infected neonates and infants^14^,^15^,^16^. Interestingly, although severe arthralgia and arthritis did occur in children, it was uncommon and usually resolved during the acute phase of disease^16^.

In this study, we studied the immune response of CHIKV infection by examining the disease manifestations and measuring the circulatory immune mediators during acute disease in a cohort of 86 children under the age of 12. Multiplex-microbead immunoassays were done and meta-analysis was further performed together with all available data from 2009 to 2014 relating to acute CHIKV infection in adults to identify unique immune markers explaining the different clinical manifestations between children and adults. Interestingly, a differential pattern was identified in the immune mediator response between pediatric patients with early viral clearance and those with prolonged viremia and joint pain.

## Results

### Clinical manifestations of a CHIKF pediatric cohort

Between October 2009 and March 2010, 108 children were recruited for this study based on their presenting symptoms during hospital admission (Table 1). Patients’ admission sera (median sampling day was 1 day post illness onset) were subjected to virus isolation and tested by CHIKV-specific PCR test. IgM serology was performed to test for DENV infection^17^. Eighty-six children were confirmed CHIKV positive with a median age of 4.86 years, ranging from 1 week to 11 years old. The length of stay was between 2–8 days. The most reported clinical manifestations of this cohort were fever, joint pain, facial flushing, skin rash (mostly erythematous, with some cases of maculopapular rash), chills, and headache (Table 1). Many of these children also suffered from loss of appetite and were not drinking well (Table 1).

Of these 86 pediatric patients, blood samples were obtained from 64 individuals during both hospital admission and at discharge (i.e. paired samples). CHIKV was still detectable by virus isolation in 34 patients at discharge and thus classified as the prolonged viremia group (Table 1). The other 30 children with no detectable CHIKV by PCR at discharge were classified as early viral clearance group (Table 1). The remaining 22 CHIKF pediatric patients were classified as the unpaired sample group as blood samples were obtained during hospital admission only.

### Profiles of immune mediators in children

**Figure 1:**
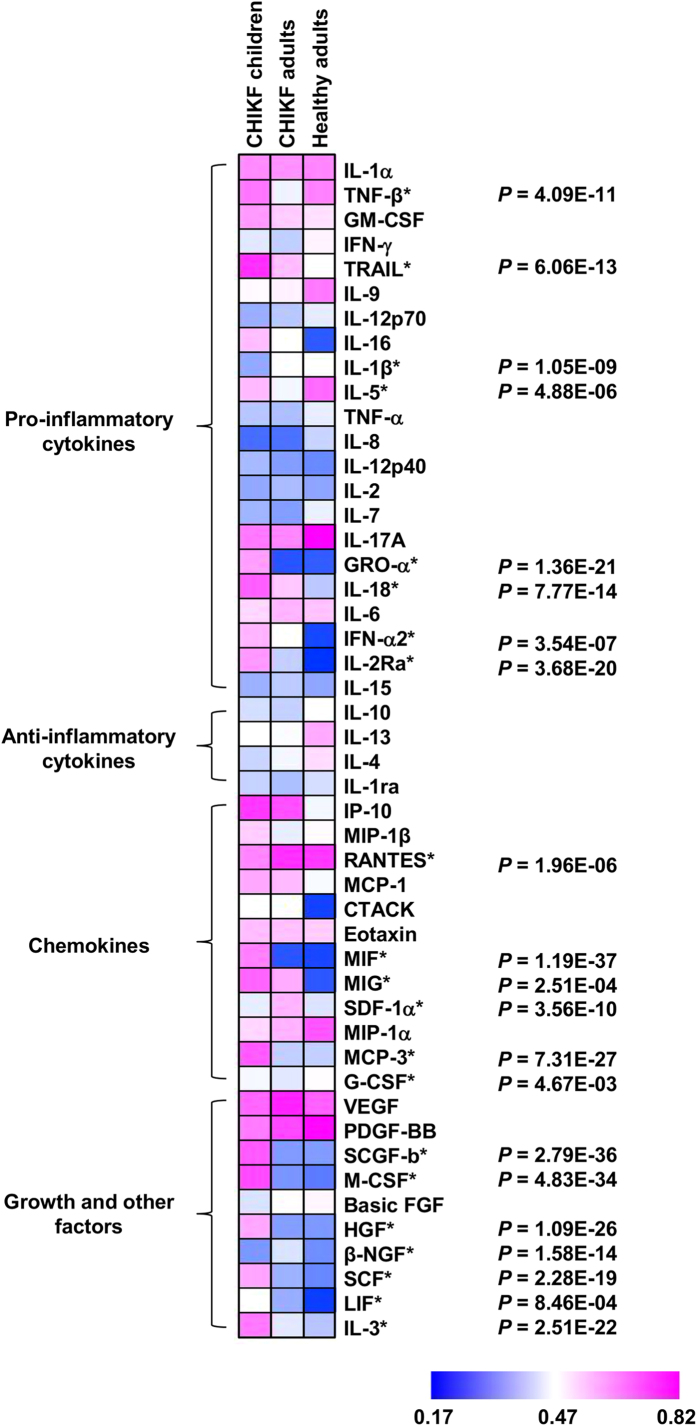
Children have higher levels of immune mediators than adults. Levels of Immune mediators (Pro-inflammatory cytokines, anti-inflammatory cytokines, chemokines and growth and other factors) during acute CHIKV infection (samples collected during the admission stage of hospitalization) from two groups of patients (86 children and 64 adults) were analyzed and presented in heat map of normalized scores. In the heat map presentation, the immune mediator concentrations were scaled between 0 and 1 for each measured immune mediator and then the average scaled value computed for each group. Blue colors represent the lowest average scaled value while pink colors represent the highest average scaled value. Immune mediators levels of healthy adults (n = 8) were included as reference point. Differences between the two groups were analyzed using Mann-Whitney *U*-test, with Bonferroni correction.

We next characterized the immune mediator profiles of acute serum samples collected from the 86 children using a multiplex-microbead immunoassay^11^. Samples from 64 adult Singapore CHIKF patients^18^,^19^ and 8 healthy adults were included in the analysis. Both groups of patients represent the population in South-East Asia, and no major difference in disease severity was observed. The pediatric cohort induced a higher level of several immune mediators when compared to the adult cohort (Fig. 1). Pro-inflammatory cytokines: TNF-β, TRAIL, IL-5, GRO-α, IL-18, IFN-α2, IL-2Ra; chemokines: MIF, MIG, MCP-3, G-CSF; growth and others: SCGF-β, M-CSF, HGF, SCF, LIF, IL-3 were significantly higher in children compared to adults (Fig. 1). However, IL-1β; RANTES, SDF-1α, and β-NGF were significantly lower in children (Fig. 1). This observation suggests that an active production of immune mediators in children could provide a strong anti-viral environment during acute disease that could result in a better clinical outcome.

### Meta-analysis of common immune mediators between adults and children

The levels of immune mediators in adult patients with CHIKV infection have been widely reported from geographically distinct cohorts and compared systematically^13^. To compare the current data set in this study, a meta-analysis was performed. Source of samples, classification of immune mediators, study selection, study exclusion, and meta-analysis were done as described^13^. Table 2 shows an overview of the expression profiles of all immune mediators that demonstrated a significant up-regulation relative to the healthy controls. Twenty-six factors: IL-2Ra, IL-6, IFN-α2, IL-16, IL-7, IL-15, IL-12, IL-18, GM-CSF, IL-1ra, IL-10, MCP-1, IP-10, MIG, SDF-1α, G-CSF (*P* < 0.01); IL-12p40, TNF-α, IL-2, IFN-γ, IL-17, IL-4, MIP-1β, RANTES, MIP-1α, FGF-β (*P* < 0.05) were found to be age-independent factors for acute CHIKV infection. Forest plots showing the significant elevated immune mediators are summarized in Supplementary Figs S1–S4. Pro-inflammatory cytokines remain as the dominant signature for acute CHIKV infection (>50%) within the four categories of immune mediators. This suggests that a common pattern of immune signature exists from CHIKV infection across ages and cohorts.

### Differential immune mediators profiles in different viremic conditions in children

**Figure 2:**
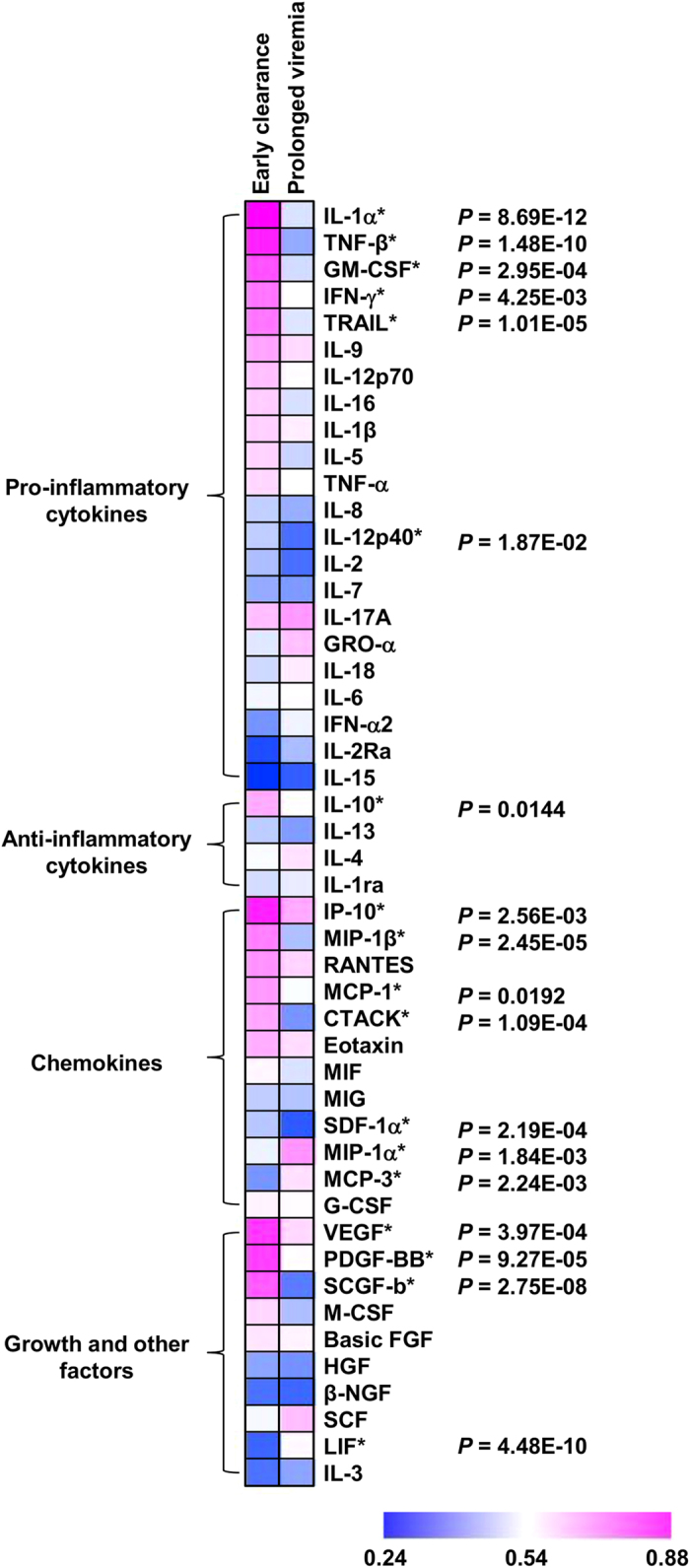
Differentially-regulated immune mediators profiles in children. Levels of immune mediators during acute CHIKV infection (samples collected during the admission stage of hospitalization) were analyzed. Patients were grouped into early viral clearance (n = 30), and prolonged viremia (n = 34) groups and compared and presented in heat map of normalized scores. In the heat map presentation, the immune mediator concentrations were scaled between 0 and 1 for each measured immune mediator and then the average scaled value computed for each group. Blue colors represent the lowest average scaled value while pink colors represent the highest average scaled value. Differences between the two groups were analyzed using Mann-Whitney *U*-test, with Bonferroni correction.

The CHIKF pediatric patients were further classified based on the viremic condition during the hospitalization period, into early viral clearance and prolonged viremia groups (Table 1). Surprisingly, a distinct profile was observed in the immune mediators between these two groups (Fig. 2). Analyzes revealed that higher levels of pro-inflammatory cytokines, chemokines, growth and other factors were obtained in the early viral clearance group (Fig. 2). Profiles of pro-inflammatory cytokines, IL-12p40, IL-1α, TNF-β, TRAIL, GM-CSF, and IFN-γ were shown to be significantly higher in the early viral clearance group than in the prolonged viremia group (Fig. 2). Interestingly, the anti-inflammatory cytokine IL-10 was also significantly higher in the early viral clearance group (Fig. 2). Profiles of chemokines, CTACK, SDF-1α, IP-10, MCP-1 and MIP-1β were significantly higher in the early viral clearance group, while MCP-3 and MIP-1α were significantly higher in the prolonged viremia group (Fig. 2). Comparatively, SCGF-β, PDGF-BB and VEGF levels were significantly higher in early viral clearance group, while LIF level was significantly lower in early viral clearance group (Fig. 2). All other markers were not significantly different between the two groups (data not shown).

**Figure 3:**
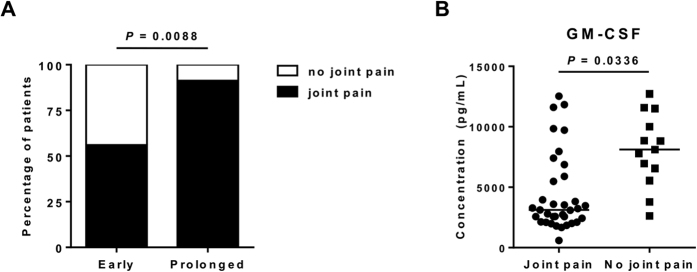
Higher proportions of patients with prolonged viremia suffered from joint pain. (**A**) Proportions of patients with joint pain within early viral clearance (n = 25) and prolonged viremia (n = 23) group were analyzed using two-sided Fisher’s exact test. GM-CSF levels were higher in patients without joint pain. (**B**) Comparison of cytokine levels between patients with (n = 35) or without joint pain (n = 13). Levels are expressed in pg/mL, horizontal lines represent median values. Two-tailed, Mann-Whitney *U*-tests with Bonferroni correction were used to evaluate differences between the two groups of patients.

To further determine if any association exists between clinical manifestations with viral clearance, the percentage of patients with different clinical parameters were compared. Our analysis revealed that facial flushing and joint pain occurred more frequently in children in the prolonged viremia group (Table 1 and Fig. 3A). Interestingly, the manifestation of joint pain in children was associated with lower levels of the pro-inflammatory cytokine GM-CSF (Fig. 3B) observed during acute CHIKV infection.

## Discussion

CHIKF affects patients of different age groups and it is important to obtain a holistic view of CHIKV-induced immune mediators from multiple cohorts of all ages. Here, the profiles of several immune regulators in CHIKV-infected pediatric patients were characterized through systematic meta-analysis in order to better define CHIKV infection in children. Clinical features such as the loss of appetite and not drinking are often linked to CHIKV infection. However, these are constitutional symptoms commonly observed in many bacterial and viral infections as well as non-communicable diseases in children^20^. Joint-specific arthralgia (specific areas including knee, wrist and small joint of the hands and feet) is a well-characterized hallmark of CHIKF during the acute phase of disease^13^. Interestingly, a significantly higher percentage of children who suffered from prolonged viremia experienced joint pain. To our knowledge, this is the first study showing a clear association between the viremic phase with arthralgia in CHIKV-infected patients.

Clinicians at the Sibu hospital observed that the overall clinical outcome observed for the majority of children in this cohort is milder than adults. Arthralgia observed in the majority of children was relatively short-lived, mild and rapidly resolved with paracetamol, which was used to provide simple analgesia and treat high fever in the study cohort. At the clinic, the cardinal signs of arthralgia (redness, swelling and increased warmth of the affected joint) were only observed in approximately 20% of the CHIKV-positive children. Indeed, the parents of affected children were more concerned about the high body temperature and skin rashes than the morbidity of arthralgia. Based on the meta-analysis data, different patterns were found in the immune factors between children and adults. Specifically, the levels of pro-inflammatory cytokines such as IL-18, IFN-α2 and IL-2Ra were much higher in children. This is interesting, as children have been reported in various studies to have low basal cytokines levels under healthy physiological conditions, although some cytokines have more complex age-dependent profiles^21^. Therefore, the high fold increase in some of the critical immune mediators such as IL-18, IFN-α2, IL-2Ra, GRO-α, MIF, MIG, MCP-3, SCGF-β, M-CSF, HGF, SCF, LIF and IL-3 from healthy to disease state could play a role in influencing disease severity in children^22^. This is in contrast to adults where the basal levels of immune mediators are generally higher, due to prior exposures to a broad range of environmental factors^23^, infections^24^, and ageing^25^.

IL-18 is one of the most important pro-inflammatory cytokines in host defense against infection, and in NK cells activation^26^. It has been established that NK cells are involved in CHIKV infections^27^. Therefore, NK cells may have a more prominent role in inflammation and homeostasis in children. Moreover, there is mounting evidence that IL-18 could be a novel therapeutic target in treating inflammatory disease^28^. On the other hand, IL-2Ra is an important indicator of T cell activation^29^ and a marker for regulatory T cells (Tregs). More recently, selective expansion of Tregs by the JES6-1 anti-IL-2 antibody complex has been shown to ameliorate experimental CHIKV-infected joint pathology^30^. Therefore, the higher level of IL-2Ra in children may indicate a larger number of Tregs in children that could alleviate joint inflammation and result in a better disease outcome^30^,^31^.

It is surprising that the manifestation of joint pain in children associated with lower levels of the pro-inflammatory cytokine GM-CSF. Since GM-CSF is often described as a key component in experimental osteoarthritis and pain development^32^ in the IL-1β pathway^32^. The high levels of GM-CSF observed here could contribute to the control of viremia through its pro-inflammatory effect and this in turn could result in an early viral clearance in this subgroup of children. This raises an interesting point as the level of GM-CSF could have a role in regulating clinical outcomes such as the development of joint pain during the acute phase of CHIKV infection even without engaging the IL-1β pathway.

Previous meta-analysis studies based on CHIKV-infected adult cohorts have defined the immune signature of mediators induced in patients suffering from acute CHIKV infection^13^. With the inclusion of new data from this pediatric cohort, a new immune signature of acute CHIKV immune mediators was derived. All categories of immune mediators exist in similar proportions, indicating that regardless of age, acute CHIKV infection is driven mainly by an inflammatory response that will in turn exert an anti-viral activity. Once again, IFN-α is significantly up-regulated during the acute phase of disease (Fig. 1) and could be a specific indicator to distinguish between CHIKV and DENV infections as the up-regulation of IFN-α is absent in DENV infection^13^.

Taken together, this is the first systematic analysis of immune mediators reported in CHIKV-infected children. The addition of this new cohort redefines the significant immune signatures that can be extended to derive a new set of biomarkers specific to acute CHIKV infection in young children (IL-18, IL-2Ra, IFN-α2, G-CSF and MIG) and adult patients (SDF-1α, RANTES). Information from the recent disease outbreaks in the Americas (from Caribbean islands to South America) will be important to provide valuable data to further validate the robustness of this new immune signature and how they can be used for differentiating CHIKV-infected patients from non-CHIKV infected patients regardless of age.

## Methods

### Ethical Approval – Children cohort from Sarawak (2009) and Adult cohort from Singapore (2008)

Written informed consent was obtained from all participants, participants’ parents or legal guardians and study was conducted according to Declaration of Helsinki principles. For the pediatric cohort, the clinical study and the use of human samples was approved by the Malaysian Ministry of Health’s Institute Review Board (KKM/NIHSEC/08/0804/P07-197). For the adult CHIKF cohort, the study was approved by the National Healthcare Group’s Domain-Specific Ethics Review Board (DSRB Reference No. B/08/026). The methods were carried out in accordance with the approved guidelines.

### Patients and Serum Collection

Sixty-four adult patients admitted with acute CHIKF to the Communicable Disease Centre at Tan Tock Seng Hospital in Singapore during the outbreak from 1 August to 23 September 2008 were included in this study^18^,^19^. Acute phase plasma specimens were collected at median 4 days post-illness onset (PIO). Clinical features definition and clinical samples were as described previously^18^,^19^. Children presented with fever or a history of fever lasting less than 7 days, one or more of the following clinical features: skin rashes, joint pain or muscle pain, and a negative blood film for malaria parasite with no apparent focal infection such as pneumonia or urinary tract infection were included into the study after obtaining informed consent from the parents or guardians. All children did not receive any medication before collection of the first blood sample and samples were taken on the day of hospital admission and at hospital discharge.

### PCR and virus isolation

All clinical specimens were screened for CHIKV using either a published^33^ or in-house (data not shown) real time RT-PCR assay. Viruses were isolated using either the C6/36 mosquito or Vero cell lines. A total of 10 Sarawak partial E1 gene nucleotide sequences from the 2009 outbreak generated in this study were analyzed together with reference sequences obtained from GenBank. A maximum likelihood phylogenetic tree was prepared and edited using the MEGA v6.06 software^34^. To assess the robustness of the tree, a bootstrap re-sampling analysis of 1000 pseudo-replicate trees was performed. A preliminary phylogenetic investigation of an approximately 500 base-pair nucleotide region of the E1 gene identified the Sarawak CHIKV as part of the East/Central/South African genotype (Supplementary Fig. S5).

### Multiplex-Microbead Immunoassay

Serum levels of immune mediators were measured using the Bio-Plex Pro™ human cytokine 21-plex (CTACK, GRO-α, HGF, IFN-α2, IL-12p40, IL-16, IL-18, IL-1α, IL-2Ra, IL-3, LIF, M-CSF, MCP-3, MIF, MIG, SCF, SCGF-β, SDF-1α, TNF-β, TRAIL, β-NGF) and 27-plex (Basic-FGF, Eotaxin, G-CSF, GM-CSF, IFN-γ, IL-10, IL-13, IL-15, IL-17A, IL-1β, IL-1Ra, IL-2, IL-4, IL-5, IL-6, IL-7, IL-8, IL-9, IP-10, IL-12p70, MCP-1, MIP-1α, MIP-1β, PDGF-BB, RANTES, TNF-α, VEGF) immunoassay kits (Bio-Rad) according to the manufacturer’s instruction. Briefly, magnetic beads were aliquoted in 96-well plates followed with addition of standards and sera from patients and control subjects. After an incubation period, plates were washed using a magnetic wash station according to manufacturer’s instructions, followed with addition of a detection antibody. Plates were incubated for a further 30 minutes and washed, followed with a final incubation of 10 minutes in the presence of streptavidin-PE. Results were acquired using the flexMAP™3D (Luminex corp.) with Luminex xPONENT^®^ software, based on standard curves plotted through a 5-parameter logistic curve setting.

### Meta-analysis

Meta-analysis (including search strategy, study selection, data extraction for comparison and statistical testing) was done previously^13^, except for the inclusion of the current data from the two cohorts. Levels of immune mediators from healthy children were obtained from published studies^21^,^35^,^36^,^37^,^38^,^39^.

### Data Analysis

Continuous variables were compared between different patient groups using non-parametric Mann-Whitney *U* test (2-tailed) analysis, with Bonferroni correction. *P*-values of < 0.05 were considered statistically significant. Hierarchical clustering was done using TM4-MeV^40^.

## Additional Information

**How to cite this article**: Simarmata, D. *et al*. Early clearance of Chikungunya virus in children is associated with a strong innate immune response. *Sci. Rep.* **6**, 26097; doi: 10.1038/srep26097 (2016).

## Supplementary Material

Supplementary Information

## Figures and Tables

**Table 1:** Demographic and characteristics of pediatric patients admitted to Sibu hospital between September 2009 and March 2010 for CHIKF.

CHIKV-positive (n = 86)^a^			
	Early clearance (n = 30)	Prolonged viremia (n = 34)	Unpaired samples (n = 22)
Age, median (range), years	3.69 (0.42–11.64)	5.93 (0.16–11.85)	5.64 (0.01–11.84)
Gender ratio (boy/girl)	1.14	1.83	1.44
Race, n (%), Chinese	8 (26.7)	8 (23.5)	6 (27.3)
Iban	13 (43.3)	20 (58.8)	11 (50)
Malay	4 (13.3)	2 (5.9)	5 (22.7)
Others	5 (16.7)	4 (11.8)	0 (0)
Length of admission, median (range), days	3 (3–6)	3 (2–8)	4 (2–7)
Fever, n (%)	30 (100)	34 (100)	22 (100)
Chills, n (%)	14 (46.7)	18 (52.9)	12 (54.5)
Facial flushing, n (%)	16 (53.3)	27 (79.4)	15 (68.2)
Skin rash, n (%)	15 (50)	20 (58.8)	13 (59.1)
Headache^b^, n (%)	13 (52)	12 (52.2)	7 (31.8)
Joint pain^b^, n (%)	14 (56)	21 (91.3)	11 (50)
Loss of appetite, n (%)	24 (80)	25 (73.5)	16 (72.7)
Not drinking, n (%)	23 (76.7)	22 (64.7)	14 (63.6)

^a^CHIKV-positive were confirmed by PCR and/or virus isolation.

^b^Clinical ‘pain’ parameters that were only recorded in patients age 2 years and older.

**Table 2:** Signature of immune mediators in patients with acute CHIKV infection.

	Pro-inflammatory cytokines (*P*-value)		Anti-inflammatory cytokines (*P*-value)		Chemokines (*P*-value)		Growth and other factors (*P*-value)	
	IL-2Ra	(6.57E-16)	IL-1ra	(1.57E-04)	MCP-1	(1.20E-08)	FGF-β	(1.88E-02)
	IL-6	(4.59E-07)	IL-10	(5.16E-04)	IP-10	(5.06E-07)		
	IFN-α2	(1.46E-06)	IL-4	(3.54E-02)	MIG	(5.31E-05)		
	IL-16	(8.83E-06)			SDF-1α	(2.07E-04)		
	IL-7	(3.48E-05)			G-CSF	(3.24E-03)		
	IL-15	(6.05E-05)			MIP-1β	(1.31E-02)		
	IL-12	(1.11E-03)			RANTES	(2.34E-02)		
	IL-18	(2.75E-03)			MIP-1α	(4.75E-02)		
	GM-CSF	(7.20E-03)						
	IL-12p40	(1.79E-02)						
	TNF-α	(1.81E-02)						
	IL-2	(2.30E-02)						
	IFN-γ	(3.07E-02)						
	IL-17	(3.14E-02)						
Average percentage of elevated immune mediators from each subcategory (%)	54		11		31		4	
