# Supplementary material for: Early clearance of Chikungunya virus in children is associated with a strong innate immune response
Source: Sci Rep. 2016 May 16;6:26097. doi: 10.1038/srep26097 (PMC4867653; doi:10.1038/srep26097)
Supplement: Supplementary Information [file srep26097-s1.pdf]

Supplementary Information

Early clearance of Chikungunya virus in children is associated with a strong innate immune response

Diane Simarmata,[1†] David Chun Ern Ng,[2†] Yiu-Wing Kam,[1†] Bernett Lee,[1] Magdline Sia Henry Sum,[3] Zhisheng Her,[1,a] Angela Chow,[4] Yee-Sin Leo,[4] Jane Cardosa,[3,b] David Perera,[3] Mong H. Ooi, [2,3,5] and Lisa F.P. Ng,[1*]

Supplementary legends

Supplementary Figure S1. Forest plots of pro-inflammatory cytokines that were significantly elevated in different CHIKF cohorts (*P* < 0.05). Dotted line represents no differences in the mean of the healthy controls and CHIKF patients. Red diamond represents the combined effect size for each mediator.

Supplementary Figure S2. Forest plots of anti-inflammatory cytokines that were significantly elevated in different CHIKF cohorts (*P* < 0.05). Dotted line represents no differences in the mean of the healthy controls and CHIKF patients. Red diamond represents the combined effect size for each mediator.

Supplementary Figure S3. Forest plots of chemokines that were significantly elevated in different CHIKF cohorts (*P* < 0.05). Dotted line represents no differences in the mean of the healthy controls and CHIKF patients. Red diamond represents the combined effect size for each mediator.

Supplementary Figure S4. Forest plots of growth and other factors that were significantly elevated in different CHIKF cohorts ($P < 0.05$). Dotted line represents no differences in the mean of the healthy controls and CHIKF patients. Red diamond represents the combined effect size for each mediator.

Supplementary Figure S5. Phylogeny of Sarawak CHIKV. A maximum likelihood tree was prepared from a 517 base-pair portion of the E1 gene. Results of this gene nucleotide sequencing work has been deposited at GenBank on 12 Nov 2015 with accession number (BS259-Sarawak-09: KU196260, BS260-Sarawak-09: KU196259, BS258-Sarawak-09: KU196261, BS239-Sarawak-09: KU196262, BS232-Sarawak-09: KU196263, BS200-Sarawak-09: KU196264, BS169-Sarawak-09: KU196265, BS166-Sarawak-09: KU196266, BS105-Sarawak-09: KU196267 and BS5-Sarawak-09: KU196268). Sequence from the closely related O'nyong-nyong virus (GenBank accession: AF192890) was used as an out-group to root the tree. CHIKV sequences from CHIKF outbreaks in Sarawak (2009) and Singapore (2007/08) are indicated with closed circle and open square labels respectively, and Indian Ocean lineage sequences are labelled with open circles. All GenBank reference sequences are identified by their accession numbers. Bootstrap values >70% are indicated at relevant branch nodes. All branch lengths are drawn to scale and a measurement of relative phylogenetic distance is provided by the scale at the bottom of the tree.

Supplementary References

1. Chaaitanya, I. K. et al. Role of proinflammatory cytokines and chemokines in chronic arthropathy in CHIKV infection. *Viral Immunol.* **24**, 265-271 (2011).

2. Chirathaworn, C., Poovorawan, Y., Lertmaharit, S. & Wuttirattanakowit, N. Cytokine levels in patients with chikungunya virus infection. *Asian Pac J Trop Med.* **6**, 631-634 (2013).
3. Chirathaworn, C., Rianthavorn, P., Wuttirattanakowit, N. & Poovorawan, Y., Serum IL-18 and IL-18BP levels in patients with Chikungunya virus infection. *Viral Immunol.* **23**, 113-117 (2010).
4. Chopra, A., Saluja, M. & Venugopalan, A., Effectiveness of chloroquine and inflammatory cytokine response in patients with early persistent musculoskeletal pain and arthritis following chikungunya virus infection. *Arthritis Rheumatol.* **66**, 319-326 (2014).
5. Chow, A et al., Persistent arthralgia Induced by chikungunya virus infection is associated with interleukin-6 and granulocyte macrophage colony-stimulating factor. *J Infect Dis* **203**, 149-157 (2010).
6. Hoarau, J. J. et al., Persistent chronic inflammation and infection by Chikungunya arthritogenic alphavirus in spite of a robust host immune response. *J Immunol.* **184**, 5914-5927 (2010).
7. Kashyap, R. S. et al., Determination of Toll-Like Receptor-Induced Cytokine Profiles in the Blood and Cerebrospinal Fluid of Chikungunya Patients. *Neuroimmunomodulation* **21**, 338-346 (2014).
8. Kelvin, A. A. et al., Inflammatory cytokine expression is associated with chikungunya virus resolution and symptom severity. *PLoS Negl Trop Dis.* **5**, e1279 (2011).
9. Lee, N. et al., Chikungunya fever, Hong Kong. *Emerg Infect Dis* **12**, 1790-1792 (2006).

10. Lohachanakul, J. et al. High concentrations of circulating interleukin-6 and monocyte chemotactic protein-1 with low concentrations of interleukin-8 were associated with severe chikungunya fever during the 2009-2010 outbreak in Thailand. *Microbiol Immunol* **56**, 134-138 (2012).
11. Ng, L. F .P et al. IL-1beta, IL-6, and RANTES as biomarkers of chikungunya severity. *PLoS One* **4**, e4261 (2009).
12. Reddy, V., Mani, R. S., Desai, A. & Ravi, V., Correlation of plasma viral loads and presence of Chikungunya IgM antibodies with cytokine/chemokine levels during acute Chikungunya virus infection. *J Med Virol.* **86**, 1393-1401 (2014).
13. Wauquier, N. et al., The acute phase of Chikungunya virus infection in humans is associated with strong innate immunity and T CD8 cell activation. *J Infect Dis.* **204**, 115-123 (2011).
14. Venugopalan, A., Ghorpade, R. P. & Chopra, A., Cytokines in Acute Chikungunya. *PLoS One* **9**, e111305 (2014).
15. Teng, T. S. et al., A Systematic Meta-analysis of Immune Signatures in Patients With Acute Chikungunya Virus Infection. *J Infect Dis.* **211**, 1925-1935 (2015).

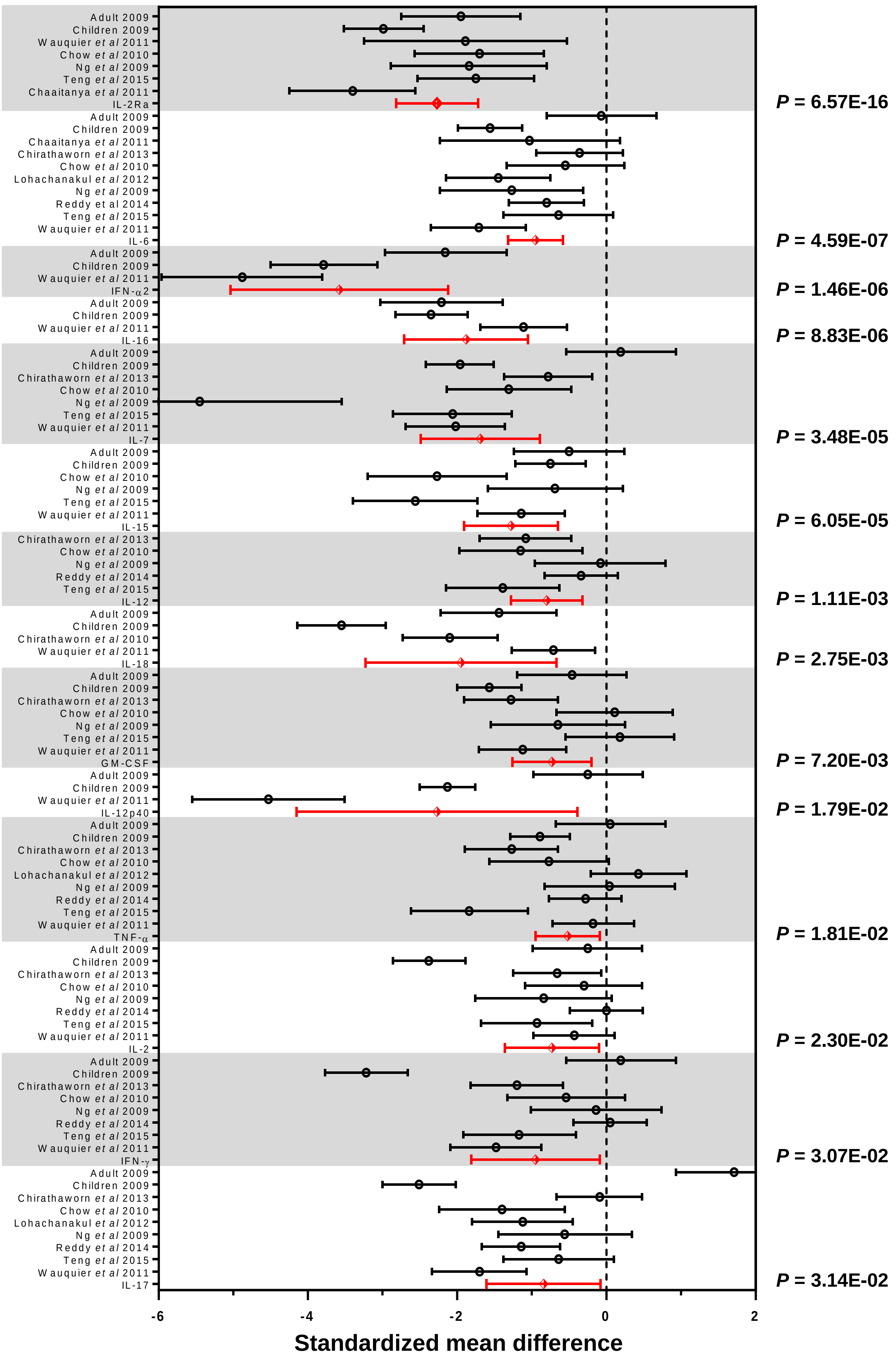

Supplementary Figure S1 Simarmata *et al*., 2016

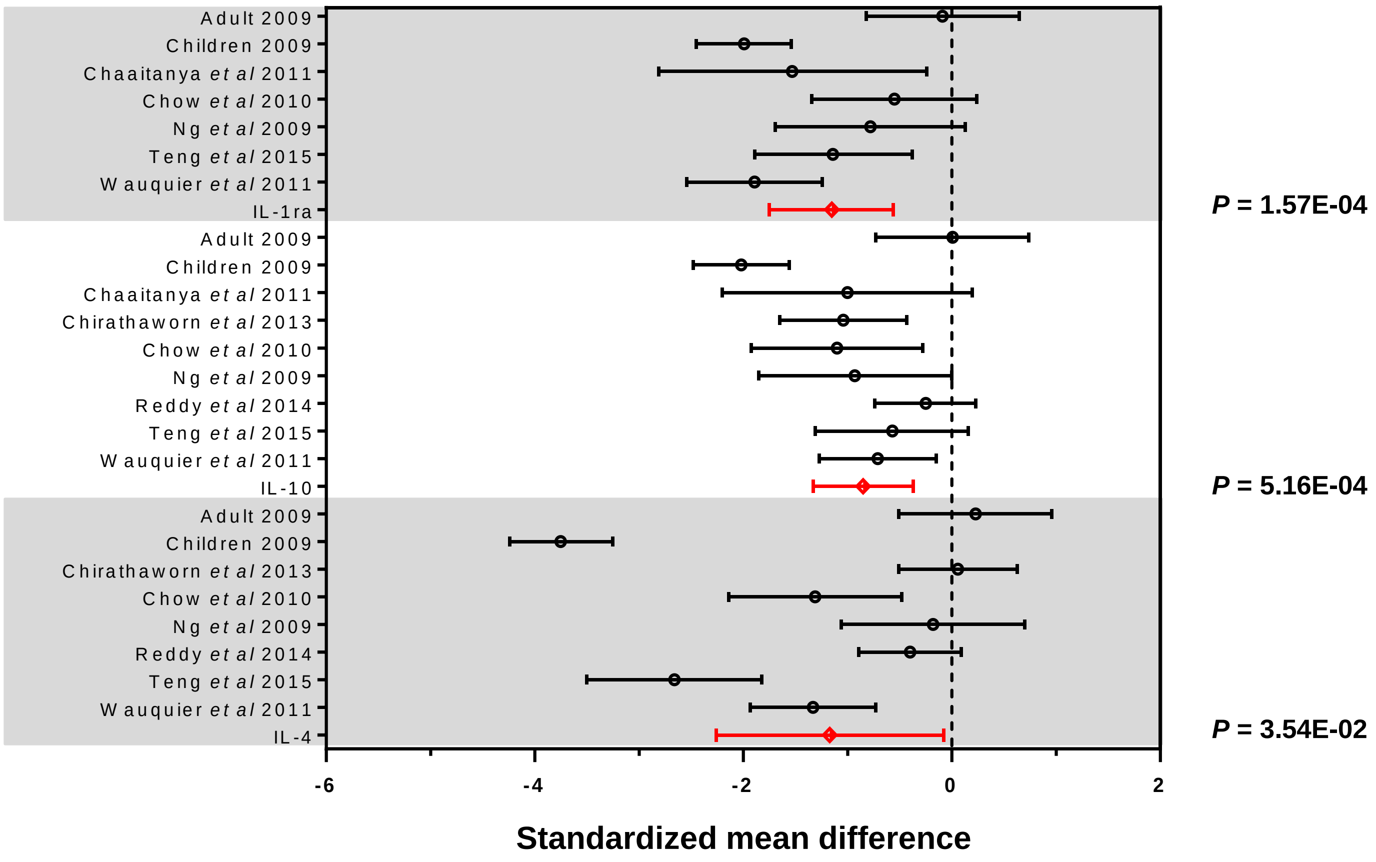

Supplementary Figure S2 Simarmata *et al*., 2016

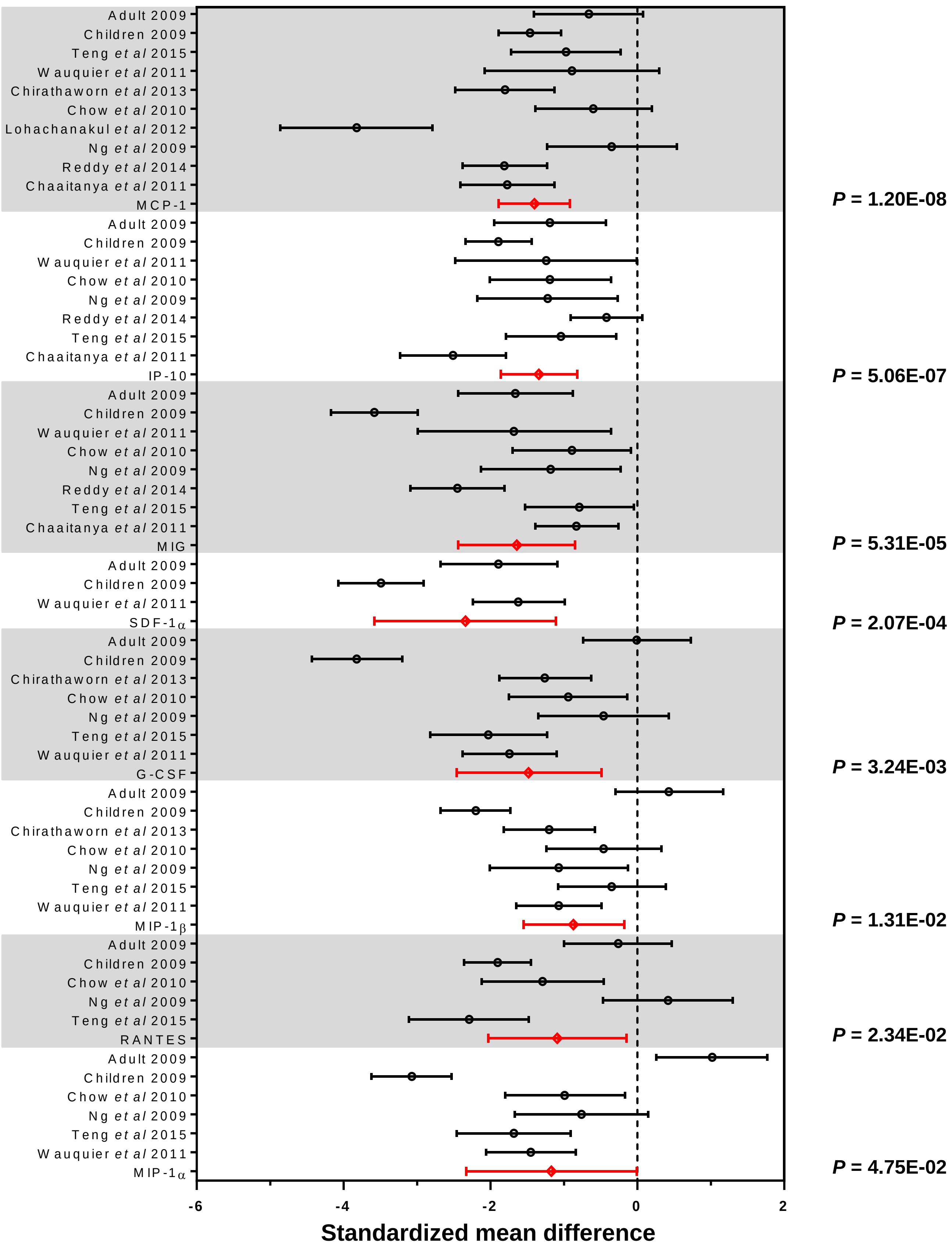

Supplementary Figure S3 Simarmata *et al*., 2016

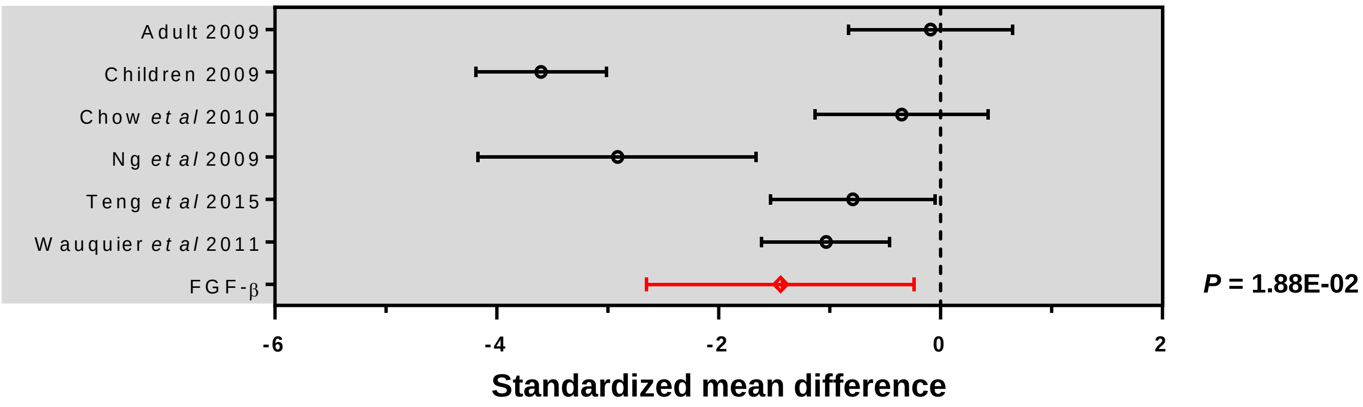

Supplementary Figure S4 Simarmata *et al*., 2016

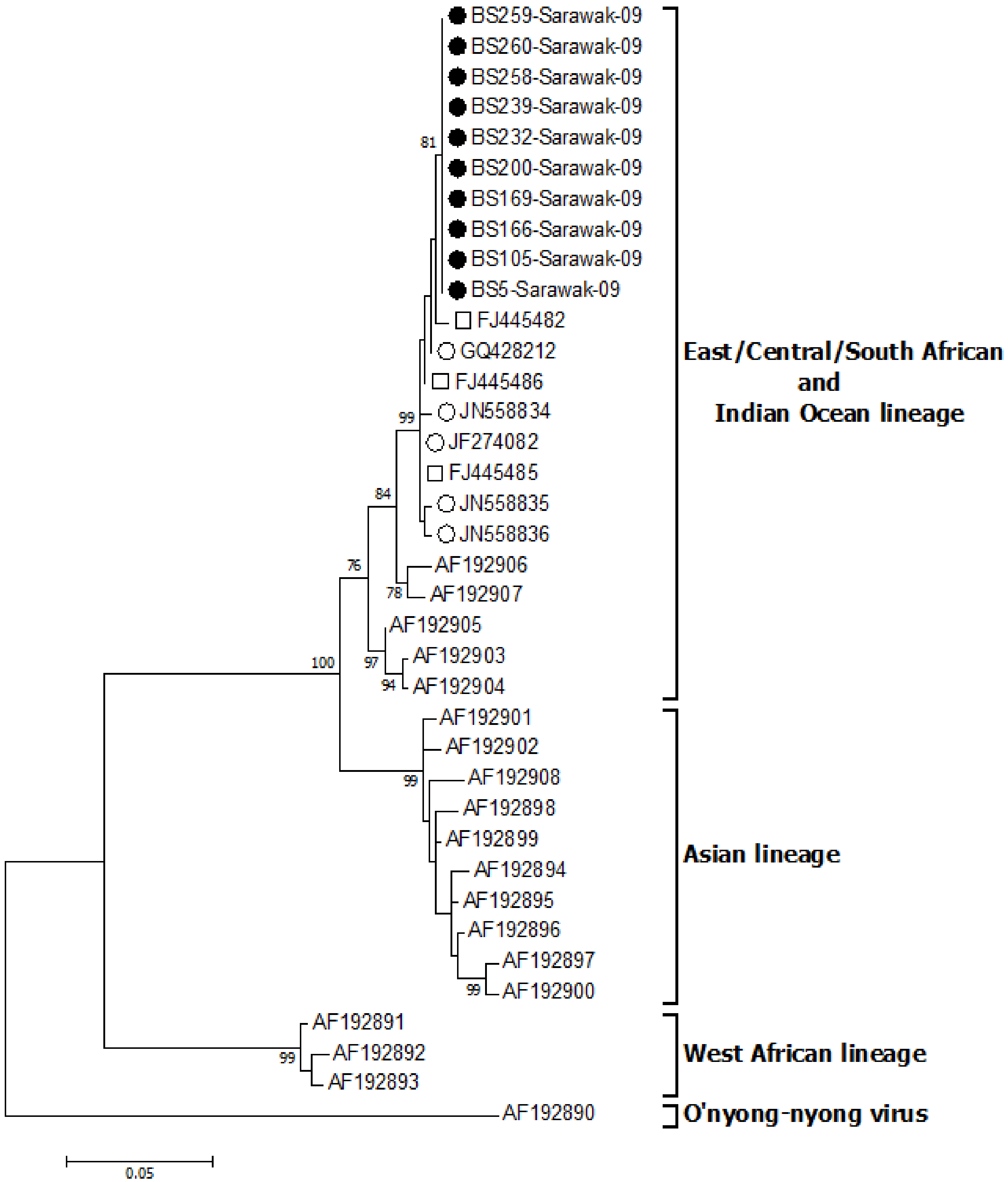

Supplementary Figure S5 Simarmata *et al*., 2016
